# Uptake of Colorectal Cancer Screening among Ontarians with Intellectual and Developmental Disabilities

**DOI:** 10.1371/journal.pone.0118023

**Published:** 2015-02-17

**Authors:** Hélène Ouellette-Kuntz, Helen Coo, Virginie Cobigo, Andrew S. Wilton

**Affiliations:** 1 Department of Public Health Sciences, Queen’s University, Kingston, Ontario, Canada; 2 Ongwanada Resource Centre, Kingston, Ontario, Canada; 3 Institute for Clinical Evaluative Sciences (ICES), Toronto, Ontario, Canada; 4 School of Psychology, University of Ottawa, Ottawa, Ontario, Canada; Leibniz Institute for Prevention Research and Epidemiology (BIPS), GERMANY

## Abstract

Under-screening for cancer may contribute to a greater disease burden in individuals with intellectual and developmental disabilities (IDD) as their life expectancy increases. In 2008, the province of Ontario launched Canada’s first population-based colorectal cancer screening program. Our objectives were to compare the proportions of Ontarians with and without IDD who have undergone colorectal cancer screening and to examine factors associated with screening uptake among Ontarians with IDD. Records for Ontario residents 50–64 years of age were linked across various administrative health and social services datasets to identify individuals with IDD and to select a random sample of the age-equivalent Ontario population without IDD as a comparison group. Logistic regression models were fit to examine the odds of screening uptake among individuals with IDD while controlling for age, sex, urban or rural residence, neighbourhood income quintile, expected use of health care resources, and being enrolled with or seeing a physician in a patient enrolment model (any of several primary care practice models designed to improve patient access and quality of care in Ontario), and to examine the association between these variables and colorectal cancer screening in the IDD population. The odds of having had a fecal occult blood test in the previous two years and being up-to-date with colorectal tests were 32% and 46% lower, respectively, for Ontarians with IDD compared to those without IDD. Being older, female, having a greater expected use of health care resources, and being enrolled with or seeing a physician in a primary care patient enrolment model were all significantly associated with higher odds of having been screened for colorectal cancer in the IDD population. These findings underscore the need for targeted interventions aimed at making colorectal cancer screening more equitable.

## Introduction

Colorectal cancer is the third most common cancer diagnosed in Canada and the second and third leading cause of cancer deaths among Canadian men and women, respectively [1]. Removal of adenomatous polyps has been shown to reduce its incidence by 76% to 90% [2]; colorectal cancer therefore presents an ideal target for early detection and prevention through screening [3]. Several screening tests are available, including flexible sigmoidoscopy, colonoscopy, the fecal occult blood test (FOBT), and, more recently, the fecal immunochemical test.

In 2008, the province of Ontario instituted the first population-based colorectal cancer screening program in Canada. The program’s goals are to increase the capacity of primary care to participate in organized colorectal cancer screening and to reduce mortality from colorectal cancer. At the time of this writing, biennial screening using the guaiac fecal occult blood test (FOBT) is recommended for individuals 50 to 74 years of age at average risk of colorectal cancer, followed by colonoscopy for those with an abnormal FOBT. For persons at increased risk because of a family history of the disease, colonoscopy is recommended beginning at age 50, or 10 years earlier than the age at which a first-degree relative was diagnosed with colorectal cancer, whichever occurs first [4].

Our group received funding from the Canadian Institutes of Health Research in 2010 to address the need for more comprehensive data to support decision-making, policy development, service provision, and research relevant to the health and well-being of Ontarians with developmental disabilities (henceforth referred to as intellectual and developmental disabilities, or IDD; under Ontario’s Services and Supports to Promote the Social Inclusion of Persons with Developmental Disabilities Act (2008), “developmental disability” is an umbrella term that includes conditions such as intellectual disability, autism spectrum disorder, and fetal alcohol spectrum disorder). Part of that work involved evaluating the extent to which the Ontario health system provides guideline-recommended primary care to adults with IDD, including preventive screening for various cancers.

While several studies have reported lower rates of breast [5,6] and cervical cancer screening [5–7] among women with intellectual and/or developmental disabilities, we are not aware of any studies that have examined participation in colorectal cancer screening among adults with IDD. As their life expectancy increases [8,9], suboptimal screening practices may contribute to a greater cancer burden in this population [10]. Our objectives, therefore, were to determine the proportion of Ontarians with IDD who have been screened for colorectal cancer; to compare their screening participation rates to those of Ontarians without IDD; and to explore whether certain sociodemographic and health care utilization characteristics are associated with screening uptake among Ontarians with IDD. In addition to providing data on an understudied issue that is important from a health care and health equity perspective, our findings will provide a baseline for monitoring trends in screening uptake among individuals with IDD, particularly as they relate to the implementation of measures to increase Ontarians’ participation in the province’s colorectal cancer screening program (discussed further on under *Ontario’s ColonCancerCheck Program*).

## Methods

Ethics approval to conduct this study was obtained from research ethics boards at Sunnybrook Health Sciences Centre and the Centre for Addiction and Mental Health (both in Toronto, Canada). Informed consent was not sought as only de-identified data (housed at Ontario’s Institute for Clinical Evaluative Sciences) were analyzed.

### Setting

Ontario has a population of 13.4 million [11]. Universal coverage of insured health services is provided to residents through a single payer, the Ontario Health Insurance Plan (OHIP). Since the early 2000s, health care delivery and modes of physician compensation in the province have undergone significant reform with the introduction of various primary care practice models designed to improve patient access and quality of care relative to the traditional fee-for-service model [12]. While a detailed description of these models is beyond the scope of this paper, a brief summary of two key elements—physician compensation and patient enrolment—will provide some useful context. Physician compensation mechanisms in Ontario are classified into two broad categories: full fee-for-service, where physicians bill OHIP a standard amount for each service they perform, and alternative payment plans. The latter category includes salaried physicians as well as those paid through blended compensation models, which offer varying levels of fee-for-service and capitation payments. In most cases, with the exception of those who work in Community Health Centres and Health Services Organizations [13], physicians compensated through alternative payment plans are required to submit shadow billings to OHIP. Shadow billings contain information about the services provided, but are used for administrative and tracking rather than reimbursement purposes.

Patient enrolment is a process whereby an individual formally agrees to receive all their primary care from a specific provider (or team), who in turn agrees to provide comprehensive primary care to that individual [14]. Patient enrolment is mandatory in some of the new primary care practice models and optional in others (regardless, these primary care practice models are also referred to as “patient enrolment models” in this paper). While all primary care physicians in Ontario receive financial incentives to conduct colorectal cancer screening, those who practice in patient enrolment models receive a larger bundle of incentives (e.g. the Colorectal Cancer Screening Preventive Care Bonus) [15].

### Ontario’s ColonCancerCheck Program

As of 2008, the list of insured health services in Ontario includes colorectal cancer screening through the province’s ColonCancerCheck Program (i.e. eligible residents do not pay for screening). Primary care providers play a pivotal role in the Program; they dispense FOBT kits or make referrals for colonoscopy, receive the results for all FOBTs they dispense, and are responsible for following up on the results of those tests. Individuals who do not have a primary care provider can get an FOBT kit through pharmacies or by calling Telehealth Ontario (a free, confidential telephone service staffed by registered nurses that provides health information and advice) [4].

In 2010, an invitation system was launched to increase participation in the ColonCancerCheck Program. Recall letters were sent to those who were due for their biennial repeat screening and invitations were sent to newly screen-eligible Ontarians. The Program’s 2010 Report also noted plans to send invitations to all eligible residents who were under-screened or who had never been screened [4].

### Study design

This was a routine-data-based study [16] in which records from a variety of administrative data sources were linked to examine colorectal cancer screening uptake among Ontario residents 50–64 years of age with and without IDD.

### Data sources

As noted in the introduction, the analysis described in this paper formed part of a larger project to examine primary care among individuals with IDD. The methods for that project, including a detailed description of the data sources, are described elsewhere [17]. Briefly, the data were accessed through Ontario’s Institute for Clinical Evaluative Sciences (ICES; www.ices.on.ca). ICES houses administrative health databases that contain information used by the provincial government for funding and reimbursement purposes. ICES’ holdings also include a registry that contains basic demographic information on all residents who have ever had an OHIP number (the Registered Persons Database), as well as provincial disease registries (including the Ontario Cancer Registry). Records for the same individuals can be linked across these datasets using unique encoded identifiers.

For the larger project, a special dataset was brought into ICES through a data sharing agreement with the Ministry of Community and Social Services, which administers the Ontario Disability Support Program (ODSP). ODSP provides income and employment supports to individuals with disabilities between 18 and 64 years of age, and collects demographic and basic diagnostic information for eligibility purposes. ODSP records from April 1, 2009 to March 31, 2010 were probabilistically linked to records in the Registered Persons Database housed at ICES. Records in the resulting dataset were then merged with and linked to those in five administrative health databases that capture the majority of Ontario residents’ encounters with the health care system. Those databases included the Canadian Institute for Health Information’s Discharge Abstracts Database (inpatient hospital discharges; information captured since 1988); the Same-day Surgery Database (information captured since 1991); the National Ambulatory Care Reporting System Database (visits to emergency departments; information captured since 2000); the Ontario Mental Health Reporting System Database (inpatient mental health bed discharges; information captured since 2005); and the OHIP Database (claims submitted by fee-for-service physicians, shadow billings submitted by most physicians compensated through alternative payment plans, and claims for laboratory services [18]; information captured since 1991). Additional linkages were made to records in the Ontario Cancer Registry, a database containing information on all Ontario residents newly diagnosed with cancer (information captured since 1964), and area-level demographic data from the 2006 Canadian Census.

### Study cohort

For the analysis described in this paper, the target population comprised Ontarians 50–64 years of age on March 31, 2010 who resided in Ontario on April 1, 2009 and were eligible for OHIP benefits from April 1, 2008 through March 31, 2010. To identify individuals with IDD, a list of ICD-9 and ICD-10 diagnostic codes was developed by reviewing codes used in previously published studies and comparing them with the codes used to meet eligibility criteria for government-funded services and supports in Ontario due to the presence of a developmental disability [17,19]. (That list of diagnostic codes can be viewed on pages 154 and 155 of the Atlas on the Primary Care of Adults with Developmental Disabilities in Ontario [17].) Individuals were considered to have IDD if a relevant diagnostic code appeared at least twice in the OHIP Database; at least once in any of the other four administrative health databases used in this study; or in either of the two diagnostic fields in the ODSP Database. Twenty percent of the Ontario population without IDD was randomly selected as a comparison group. Random sampling reduces computer processing time while still ensuring sufficiently large numbers for the analysis [5,17].

We applied similar exclusion criteria to those used by the ColonCancerCheck Program for its “up-to-date with colorectal tests” indicator [4]: missing or invalid health insurance number, date of birth, sex, or postal code; diagnosed prior to April 1, 2009 with colorectal cancer (ICD-9 code 153 (excluding 153.5), 154.0, or 154.1; or ICD-0–3 code C18 (excluding C18.1), C19, C20, or C26.0) in the Ontario Cancer Registry); fee code for total colectomy (S169, S170, or S172) prior to April 1, 2009 in the OHIP Database; or Q142A code in the OHIP Database from April 1, 2008 to March 31, 2010. The latter code may be submitted by physicians to indicate that a patient meets criteria for being excluded from the colorectal cancer screening target population for reasons such as history of malignant bowel disease or other disease requiring regular colonoscopies for surveillance purposes [20].

### Participation in colorectal cancer screening

Two of the ColonCancerCheck Program indicators were used to examine uptake of colorectal cancer screening [4]. The first, FOBT participation, was defined as at least one FOBT in the previous two years; this was determined by searching the OHIP Database from April 1, 2008–March 31, 2010 for relevant physician or laboratory billings (G004, L179, or L181). The second indicator, up-to-date with colorectal tests, was defined as one of the following: FOBT in the previous two years; sigmoidoscopy (fee code Z580 in the OHIP Database) in the previous five years (April 1, 2005-March 31, 2010); or colonoscopy (fee code Z555 +/- other E codes in the OHIP Database) in the previous ten years (April 1, 2000-March 31, 2010).

### Sociodemographic and health care utilization characteristics

In addition to age on March 31, 2010, we captured or derived other variables that have been shown in previous studies to be related to the uptake of cancer screening in different populations, and which were available in the data sources. Those variables included the individual’s sex [18,21]; urban or rural residence [18,22], based on Statistics Canada definitions for the 2006 census population [17]; neighbourhood income quintile [18,23], which was derived by mapping individuals’ postal codes as of April 1, 2009 to area-level income data from the 2006 census [17]; resource utilization band [5], a validated measure derived from an individual’s age, sex, and diagnostic information that is used to characterize his or her morbidity level and corresponding expected use of health care resources according to one of six categories, ranging from “Non-users” (lowest expected use of health care resources) to “Very high morbidity” (highest expected use of health care resources) [24]; and enrolment with or seeing a physician in a primary care patient enrolment model [23] as of April 1, 2009.

### Analysis

All analyses were done by an ICES staff member using SAS 9.3 (Cary, NC). Frequency distributions were generated to describe screening uptake between those with and without IDD, stratified by sociodemographic and health care utilization characteristics. Logistic regression models were fit to examine the unadjusted and adjusted odds of colorectal cancer screening uptake among Ontarians with IDD. Additional logistic regression models were fit to assess, within the cohort of Ontarians with IDD, which sociodemographic and health care utilization characteristics were associated with higher odds of having been screened for colorectal cancer.

## Results

Table 1 provides the sociodemographic and health care utilization characteristics of the study cohort stratified by IDD status, and the proportions who 1) had an FOBT in the previous two years, and 2) were up-to-date with colorectal tests as of March 31, 2010. Compared to those without IDD, proportionally more individuals with IDD were 50–59 years of age and male, lived in rural or low-income areas, and were characterized as having high or very high morbidity (i.e. greater expected use of health care resources). Fewer were enrolled with or seeing a physician in a primary care patient enrolment model. Overall, 18.3% of Ontarians with IDD had an FOBT in the previous two years and 32.0% were up-to-date with colorectal tests; the corresponding proportions for those without IDD were 26.4% and 47.2%, respectively (Table 2). After controlling for the other study variables, the odds of having had an FOBT in the previous two years were 32% lower for Ontarians with IDD; similarly, their odds of being up-to-date with colorectal tests were about half those of Ontarians without IDD (Table 2).

**Table 1:** Sociodemographic and health care utilization characteristics of Ontarians 50–64 years of age with and without intellectual and developmental disabilities, and proportions who participated in colorectal cancer screening.

	Ontarians with intellectual and developmental disabilities (n = 15,791)			Ontarians without intellectual and developmental disabilities (n = 791,792)		
	n (%)	Fecal occult blood test in previous two years*, %	Up-to-date with colorectal tests^†^, %	n (%)	Fecal occult blood test in previous two years*, %	Up-to-date with colorectal tests^†^, %
**Age group, years**						
50–54	6769 (42.9)	16.6	28.8	309,199 (39.1)	22.1	39.5
55–59	5296 (33.5)	19.2	33.1	259,293 (32.7)	27.4	49.4
60–64	3726 (23.6)	20.2	36.1	223,300 (28.2)	31.3	55.1
**Sex**						
Male	8505 (53.9)	17.0	29.7	390,964 (49.4)	24.1	44.2
Female	7286 (46.1)	19.8	34.6	400,828 (50.6)	28.7	50.1
**Residence**						
Rural	2982 (18.9)	17.1	31.3	105,354 (13.3)	23.4	44.9
Urban	12,809 (81.1)	18.6	32.1	686,438 (86.7)	26.9	47.5
**Neighbourhood income quintile**						
1 (lowest)	5320 (33.7)	18.0	30.4	139,167 (17.6)	24.4	41.0
2	3332 (21.1)	17.8	31.4	152,100 (19.2)	26.9	44.7
3	2609 (16.5)	20.5	34.7	154,355 (19.5)	27.4	47.2
4	2365 (15.0)	18.3	33.0	164,262 (20.7)	27.6	49.6
5 (highest)	1956 (12.4)	18.7	34.4	172,159 (21.7)	26.7	53.3
Missing	209 (1.3)	3.8	14.4	9749 (1.2)	8.2	23.5
**Resource utilization band** ^**‡**^						
Non-users	1085 (6.9)	3.9	5.8	61,234 (7.7)	7.5	14.0
Healthy users	370 (2.3)	12.2	15.7	30,055 (3.8)	21.3	31.9
Low morbidity	1607 (10.2)	16.6	22.7	115,351 (14.6)	24.2	37.3
Moderate morbidity	7876 (49.9)	20.3	33.7	448,143 (56.6)	29.6	52.0
High morbidity	2814 (17.8)	20.0	39.3	98,213 (12.4)	28.6	58.7
Very high morbidity	2039 (12.9)	18.3	39.4	38,796 (4.9)	24.5	55.1
**Enrolled with or seeing a physician in a primary care patient enrolment model**						
No	5394 (34.2)	10.5	22.2	220,568 (27.9)	14.3	31.9
Yes	10,397 (65.8)	22.4	37.0	571,224 (72.1)	31.1	53.1

*April 1, 2008-March 31, 2010

^†^ Fecal occult blood test in previous two years (April 1, 2008-March 31, 2010); sigmoidoscopy in previous five years (April 1, 2005-March 31, 2010); or colonoscopy in previous ten years (April 1, 2000-March 31, 2010)

^‡^ A validated measure of morbidity that defines an individual’s expected use of health care resources [24]

**Table 2:** Odds ratios for uptake of colorectal cancer screening among Ontarians 50–64 years of age with intellectual and developmental disabilities.

	Fecal occult blood test in previous two years*				Up-to-date with colorectal tests†			
	n	%	Unadjusted OR (95% CI)	Adjusted^‡^ OR (95% CI)	n	%	Unadjusted OR (95% CI)	Adjusted^‡^ OR (95% CI)
**Intellectual and developmental disability**								
Yes	2890	18.3	0.62 (0.60–0.65)	0.68 (0.65–0.71)	5049	32.0	0.53 (0.51–0.54)	0.54 (0.52–0.56)
No	209,276	26.4	1.00	1.00	373,455	47.2	1.00	1.00

CI: Confidence interval

OR: Odds ratio

* April 1, 2008-March 31, 2010

† Fecal occult blood test in previous two years (April 1, 2008-March 31, 2010); sigmoidoscopy in previous five years (April 1, 2005-March 31, 2010); or colonoscopy in previous ten years (April 1, 2000-March 31, 2010)

^‡^ Models adjusted for age as of March 31, 2010, sex, urban or rural residence, neighbourhood income quintile, resource utilization band [24], and whether enrolled with or seeing a physician in a primary care patient enrolment model

Table 3 provides unadjusted and adjusted odds ratios for the sociodemographic and health care utilization characteristics of the population with IDD in relation to having had an FOBT in the previous two years. Older individuals, women, those in any resource utilization band above the reference category of “Non-users,” and those enrolled with or seeing a physician in a patient enrolment model were significantly more likely to have had an FOBT. Living in an urban area was not significantly associated with FOBT participation in this population. Similar results were observed for the association between these same characteristics and being up-to-date with colorectal tests (Table 4).

**Table 3:** Frequency distributions and odds ratios for having had a fecal occult blood test in previous two years among Ontarians 50–64 years of age with intellectual and developmental disabilities, by sociodemographic and health care utilization characteristics.

	Fecal occult blood test in previous two years* (n = 2890)		No fecal occult blood test in previous two years* (n = 12,901)		Unadjusted OR (95% CI)	Adjusted^†^ OR (95% CI)
	n	%	n	%		
**Age group, years**						
60–64	753	26.1	2973	23.0	1.28 (1.16–1.41)	1.29 (1.16–1.43)
55–59	1016	35.2	4280	33.2	1.20 (1.09–1.31)	1.21 (1.10–1.33)
50–54	1121	38.8	5648	43.8	1.00	1.00
**Sex**						
Female	1444	50.0	5842	45.3	1.21 (1.11–1.31)	1.13 (1.04–1.23)
Male	1446	50.0	7059	54.7	1.00	1.00
**Residence**						
Urban	2381	82.4	10,428	80.8	1.11 (1.00–1.23)	1.10 (0.99–1.23)
Rural	509	17.6	2473	19.2	1.00	1.00
**Neighbourhood income quintile** ^**‡**^						
5 (highest)	365	12.7	1591	12.5	1.05 (0.92–1.20)	1.01 (0.88–1.15)
4	432	15.0	1933	15.2	1.02 (0.90–1.16)	0.99 (0.87–1.13)
3	536	18.6	2073	16.3	1.18 (1.05–1.33)	1.14 (1.01–1.29)
2	593	20.6	2739	21.6	0.99 (0.88–1.11)	0.95 (0.85–1.07)
1 (lowest)	956	33.2	4364	34.4	1.00	1.00
**Resource utilization band** ^**‖**^						
Very high morbidity	374	12.9	1665	12.9	5.58 (4.02–7.75)	3.51 (2.51–4.89)
High morbidity	563	19.5	2251	17.4	6.21 (4.50–8.57)	3.86 (2.78–5.36)
Moderate morbidity	1600	55.4	6276	48.6	6.33 (4.63–8.66)	3.95 (2.87–5.43)
Low morbidity	266	9.2	1341	10.4	4.93 (3.52–6.89)	3.31 (2.36–4.65)
Healthy users	45	1.6	325	2.5	3.44 (2.22–5.33)	2.50 (1.60–3.89)
Non-users	42	1.5	1043	8.1	1.00	1.00
**Enrolled with or seeing a physician in a primary care patient enrolment model**						
Yes	2324	80.4	8073	62.6	2.46 (2.23–2.71)	2.13 (1.93–2.36)
No	566	19.6	4828	37.4	1.00	1.00

CI: Confidence interval

OR: Odds ratio

*April 1, 2008-March 31, 2010

^†^Models adjusted for all variables shown in table

^‡^209 missing values

^‖^A validated measure of morbidity that defines an individual’s expected use of health care resources [24]

**Table 4:** Frequency distributions and odds ratios for being up-to-date with colorectal tests as of March 31, 2010 among Ontarians 50–64 years of age with intellectual and developmental disabilities, by sociodemographic and health care utilization characteristics.

	Up-to-date with colorectal tests* (n = 5049)		Not up-to-date with colorectal tests* (n = 10,742)		Unadjusted OR (95% CI)	Adjusted^†^ OR (95% CI)
	n	%	n	%		
**Age group, years**						
60–64	1345	26.6	2381	22.2	1.40 (1.28–1.52)	1.36 (1.25–1.49)
55–59	1754	34.7	3542	33.0	1.22 (1.13–1.32)	1.21 (1.12–1.31)
50–54	1950	38.6	4819	44.9	1.00	1.00
**Sex**						
Female	2523	50.0	4763	44.3	1.26 (1.17–1.34)	1.15 (1.07–1.23)
Male	2526	50.0	5979	55.7	1.00	1.00
**Residence**						
Urban	4116	81.5	8693	80.9	1.04 (0.95–1.13)	1.02 (0.93–1.11)
Rural	933	18.5	2049	19.1	1.00	1.00
**Neighbourhood income quintile** ^**‡**^						
5 (highest)	672	13.4	1284	12.2	1.20 (1.07–1.34)	1.16 (1.04–1.30)
4	780	15.5	1585	15.0	1.13 (1.02–1.25)	1.10 (0.99–1.23)
3	905	18.0	1704	16.1	1.22 (1.10–1.34)	1.19 (1.07–1.31)
2	1045	20.8	2287	21.7	1.05 (0.95–1.15)	1.03 (0.93–1.13)
1 (lowest)	1617	32.2	3703	35.1	1.00	1.00
**Resource utilization band** ^**‖**^						
Very high morbidity	804	15.9	1235	11.5	10.56 (8.07–13.83)	7.48 (5.68–9.85)
High morbidity	1105	21.9	1709	15.9	10.49 (8.04–13.68)	7.40 (5.64–9.71)
Moderate morbidity	2655	52.6	5221	48.6	8.25 (8.37–10.68)	5.80 (4.45–7.55)
Low morbidity	364	7.2	1243	11.6	4.75 (3.59–6.29)	3.56 (2.68–4.74)
Healthy users	58	1.1	312	2.9	3.02 (2.07–4.40)	2.39 (1.63–3.51)
Non-users	63	1.2	1022	9.5	1.00	1.00
**Enrolled with or seeing a physician in a primary care patient enrolment model**						
Yes	3850	76.3	6547	60.9	2.06 (1.91–2.22)	1.71 (1.58–1.85)
No	1199	23.7	4195	39.1	1.00	1.00

CI: Confidence interval

OR: Odds ratio

*Fecal occult blood test in previous two years (April 1, 2008-March 31, 2010); sigmoidoscopy in previous five years (April 1, 2005-March 31, 2010); or colonoscopy in previous ten years (April 1, 2000-March 31, 2010)

^†^Models adjusted for all variables shown in table

^‡^209 missing values

^‖^A validated measure of morbidity that defines an individual’s expected use of health care resources [24]

Certain strata of neighbourhood income had a weak but significant association with screening uptake. Ontarians with IDD from neighbourhoods that fell in the middle income quintile were more likely to have had an FOBT than those living in the poorest neighbourhoods, while those living in the highest or middle income quintiles were significantly more likely to be up-to-date with colorectal tests compared to those in the poorest neighbourhoods.

## Discussion

The absolute difference between the proportions of Ontarians with and without IDD who had an FOBT in the previous two years was 8.1% (Table 2). While this is less of a disparity than the differences observed between these groups in terms of cervical and breast cancer screening uptake (32.5% and 18.3%, respectively [5]), that may be due to the low participation in colorectal cancer screening overall: only 26.3% of the study cohort had an FOBT in the previous two years (data not shown in tabular format). The absolute difference between the groups in the “up-to-date with colorectal tests” indicator was larger, at 15.2% (Table 2). While these absolute differences do not take into account the unequal distribution between the groups of characteristics that may be associated with screening participation (e.g. age, sex; Table 1), Ontarians with IDD were still significantly less likely to have participated in colorectal cancer screening after controlling for various sociodemographic and health care utilization characteristics (Table 2).

The term “equity” encompasses notions of fairness and social justice [25]. When a disparity in health care exists among groups with equal needs—in the case of colorectal cancer screening, “need” at a population level is based on age—it indicates a lack of horizontal equity [21]. Our findings, then, reveal a horizontal inequity in access to colorectal cancer screening among Ontarians with IDD. Moreover, significant disparities in screening uptake were also observed within subgroups of the IDD population, in that older individuals, women, those who were categorized in a resource utilization band above “Non-users,” and those enrolled with or seeing a physician in a primary care patient enrolment model were more likely to have been screened (Tables 3 and 4). In contrast, while one study reported that Ontarians living in rural areas were less likely to have been screened for colorectal cancer than urban residents [18], this was not a significant predictor of screening uptake in the IDD population (Tables 3 and 4). The findings for neighbourhood income are more difficult to interpret: some of the results were significant, but there was no clear linear gradient when moving from lower- to higher-income neighbourhoods (Tables 3 and 4). Most of these findings are similar to the patterns observed in the unadjusted proportions for those without IDD (Table 1).

As previously noted, the ColonCancerCheck Program has launched an invitation system in an effort to increase screening uptake among age-eligible Ontarians [4]. Whether that will have any impact on individuals with IDD is unknown; a 2009 report issued by Cancer Care Ontario noted that client reminders are an effective population-based intervention to increase uptake of breast, cervical and colorectal cancer screening, but also acknowledged the lack of evidence on the effectiveness of interventions for different population groups [26].

In order to implement interventions that effectively address inequities in colorectal cancer screening—between those with and without IDD, and among subgroups of both populations—it is crucial to first identify the barriers to, and facilitators of, access to screening. A review article published in 2010 identified a number of patient and system barriers and facilitators [21]. While some of these (e.g. embarrassment) may apply across population groups, individuals with IDD face unique barriers [10,27]. In the case of colorectal cancer screening, the FOBT requires at-home collection of two specimens each from three separate stools. (Note that a pilot project is now underway in Ontario to investigate the feasibility of replacing the FOBT with the fecal immunochemical test; the latter has been found to increase screening participation rates, likely because it requires fewer samples and the collection method is simpler [28].) Most adults with IDD will need to rely on others to support them in these tasks. Thus, caregivers’ attitudes, knowledge and skills need to be examined in terms of their potential role in facilitating colorectal cancer screening among individuals with IDD.

That same review reported that lack of physician recommendation is the primary system barrier to colorectal cancer screening [21]. Our study was not designed to determine the barriers to, and facilitators of, access to colorectal cancer screening among persons with IDD. Given the design of Ontario’s colorectal cancer screening program (described earlier under *Ontario’s ColonCancerCheck Program*), however, it seems reasonable to assume that the supply side plays a key role in screening uptake. Some of our findings indirectly support this. For example, we observed a strong association between higher morbidity/expected use of health care resources and participation in colorectal cancer screening (albeit without a consistent linear gradient for FOBT participation; see Table 3), which suggests that physicians engage in opportunistic screening of individuals who have greater contact with the health care system. Moreover, being enrolled with or seeing a physician in a primary care patient enrolment model was positively associated with screening participation among Ontarians with IDD (Tables 3 and 4). We could not determine, from the data available, which characteristics of patient enrolment models promote screening, but one hypothesis relates to financial incentives: an increase in screening uptake was observed prior to the 2008 launch of the ColonCancerCheck Program, which may have been at least partly attributable to the province’s introduction in 2006 of financial incentives for colorectal cancer screening [18]. As stated earlier, physicians in patient enrolment models receive greater financial incentives to conduct colorectal cancer screening than physicians who practice under the traditional fee-for-service model [15].

The preceding two paragraphs allude to the potentially important role caregivers and physicians play in colorectal cancer screening uptake among individuals with IDD. From that perspective, it might be useful to evaluate caregivers’ and physicians’ knowledge regarding colorectal cancer risk in this population. Individuals with Down syndrome—one of the most common known causes of intellectual disability [29]—have a lower risk of solid tumours [30], including colorectal cancer [31], than the general population. That fact may influence beliefs about colorectal cancer risk among the broader population of individuals with IDD, and hence their need for screening. Caregivers and physicians need to be aware that those with Down syndrome constitute a unique group within the IDD population [31], and that women with intellectual disabilities may actually be at increased risk of colorectal cancer compared to women in the general population [32].

As previously noted, in Ontario colonoscopy is the recommended screening procedure only for those at increased risk of colorectal cancer. In other jurisdictions, it is more commonly used for screening purposes [33]. Some individuals with IDD require sedation for minor surgery, which could present an opportunity to perform a screening colonoscopy, depending on the facility and degree of coordination and cooperation among providers [27]. However, colonoscopies present some risks and require complete cleansing of the colon. One American study reported a high rate (46%) of inadequate preparation for the procedure among individuals with IDD [31]. Thus, it is unclear whether colonoscopy could form a viable first-line screening tool to increase screening uptake among adults with IDD. If Ontario does adopt the fecal immunochemical test for colorectal cancer screening, that test’s simpler collection method may increase screening uptake in the IDD population (and with the baseline data generated by the current study, we will be able to evaluate whether this is the case in a future analysis).

A notable strength of this study is the province-wide coverage of the data sources used. For this reason, and because the OHIP Database captures fee-for-service claims and shadow billings as well as claims for FOBTs processed in laboratories (excluding specimens processed in hospitals), it is likely we were able to identify most of the age-eligible population who have been screened. Moreover, the use of administrative data may be a more accurate method of measuring screening uptake compared to surveys, as certain groups may over-report participation in cancer screening [34]. Nevertheless, we acknowledge certain limitations related to the measurement of screening uptake: a small proportion of FOBTs may have been done for diagnostic rather than screening purposes [4,18], and we could not identify and exclude those from the analysis; FOBTs analyzed in hospital laboratories would not have been captured [4,18]; some of those captured may have included kits that were rejected at the time of testing [4,18]; we were unable to identify individuals at increased risk of colorectal cancer and exclude them from the analysis; and a small number of colonoscopies may not have been captured in the administrative data [4].

A further limitation is that not all variables that are potentially important in terms of screening uptake are captured in the administrative datasets we used. These include factors such as ethnicity and individual measures of socioeconomic status [21]. Living arrangements (e.g. group home, independent living) could also be an important determinant of screening participation among individuals with IDD, but that information was not available in the data sources. Moreover, identifying individuals with IDD is a recognized challenge for researchers [35]. In this study, IDD status was defined based on the presence of ICD-9 and ICD-10 diagnostic codes in administrative datasets, and those administrative diagnoses have not been validated using a clinical reference standard. Furthermore, it is likely that administrative data fail to identify the entire population of individuals with IDD [36,37]. If some individuals were misclassified as “non-IDD” in our cohort, and if they have similar screening participation rates to those who were accurately classified as having IDD, it could mean that disparities in screening uptake may be even larger than reported here.

As a final note, this study focused on participation in the initial stage of colorectal cancer screening. However, equal access to all components of the screening process is necessary to achieve true equity in health care. Those other components include follow-up evaluation in the case of a positive screening test result and subsequent treatment, if warranted. A lack of availability and access to data is an acknowledged difficulty in tracking inequities across the entire screening process [21].

## Conclusion

Even in systems that provide universal access to health care, certain groups encounter barriers that hinder their utilization of services [21]. Equity of access to colorectal cancer screening programs has been studied in various population groups, including those defined by socioeconomic status and ethnicity (reviewed by Javanparast et al., 2010 [21]), but, to our knowledge, this is the first study to examine this issue in adults with IDD. The Canadian Consensus Guidelines on the Primary Care of Adults with Developmental Disabilities recommends regular screening for colorectal cancer in adults older than 50 years [38]. Despite this recommendation, our findings reveal low uptake of colorectal cancer screening among Ontarians with IDD. While similar sociodemographic and health care utilization characteristics appeared to be associated with screening participation among individuals with and without IDD in our study, there are likely reasons for lower uptake that are unique to the former group. This underscores the need to identify the barriers to and facilitators of colorectal cancer screening among individuals with IDD, and to develop effective interventions to increase screening uptake in this population.
